# Küttner's tumor of the sub-mandibular gland associated with fibrosclerosis and follicular hyperplasia of regional lymph nodes: a case report

**DOI:** 10.1186/1752-1947-5-121

**Published:** 2011-03-29

**Authors:** Yumi Mochizuki, Ken Omura, Kou Kayamori, Kei Sakamoto, Hiroaki Shimamoto, Akira Yamaguchi

**Affiliations:** 1 Oral and Maxillofacial Surgery, Department of Oral Restitution, Division of Oral Health Sciences, Graduate School, Tokyo Medical and Dental University, 1-5-45 Yushima, Bunkyo-ku, Tokyo 113-8549, Japan; 2 Molecular Pathology, Department of Oral Restitution, Division of Oral Health Sciences, Graduate School, Tokyo Medical and Dental University, 1-5-45 Yushima, Bunkyo-ku, Tokyo 113-8549, Japan

## Abstract

**Introduction:**

Küttner's tumor is characterized through histology by peri-ductal fibrosis, dense lymphocytic infiltration with lymphoid follicles, loss of acini, and occasional marked sclerosis of the salivary gland. On occasion, Küttner's tumor can be difficult to distinguish from malignant neoplasm.

**Case presentation:**

A 58-year-old Japanese man was referred to our hospital with a three-month history of a painless swollen mass in the right sub-mandibular region. Histological findings revealed both lymphoid follicles with reactive germinal centers and variously sized lymphoid follicle-like nodules without definitive germinal centers or mantle zones. B-cells of similar size and shape occupied the lymphoid follicle-like nodules and stained positive for B-cell lymphoma. These cells were detected in the polyclonal B-cells by flow cytometric analysis and tested negative for CD10. Unusual B-cell proliferation was observed, but as there was no definitive evidence of B-cell lymphoma, the lesion was diagnosed as Küttner's tumor.

**Conclusion:**

We report on a rare case of Küttner's tumor associated with fibrosclerosis and atypical lymphoid hyperplasia in both the sub-mandibular gland and regional lymph nodes. Although more cases need to be investigated, our findings might be helpful to further studies seeking to clarify the etiology of idiopathic sclerosing lesions arising in the organs and regional lymph nodes.

## Introduction

Küttner's tumor, or sclerosing sialoadenitis, is characterized through histology by peri-ductal fibrosis, dense lymphocytic infiltration with lymphoid follicles, loss of the acini and, occasionally, marked sclerosis of the salivary gland [1]. Here we report a rare case of Küttner's tumor of the sub-mandibular gland associated with regional lymph nodal adenopathy. Histological examination revealed that the architecture of both the sub-mandibular gland and lymph nodes showed marked fibrocollagenous changes and variously sized lymphoid follicle-like nodules with atypical B-cell proliferations. Recently, Küttner's tumor has been regarded as an immunoglobulin G4 (IgG4) -related idiopathic sclerosing lesion, a lesion which is frequently associated with regional lymph nodal adenopathy [2]. However, our case had no definitive findings of an IgG4-related idiopathic sclerosing lesion.

The literature reports a few cases of regional lymph nodal adenopathy in Küttner's tumor of the sub-mandibular gland [3]. However, to the best of our knowledge, the present case of broad fibrosclerosing and atypical lymphoid hyperplasia in both the sub-mandibular gland and regional lymph nodes is very rare. We discuss the characteristics and differential diagnostic problems of Küttner's tumor.

## Case presentation

A 58-year-old Japanese man was referred to our hospital with a three-month history of a painless swollen mass in his right sub-mandibular region. Physical examination revealed an elastic hard mass in his right sub-mandibular gland. The gland was not fixed to adjacent tissue. Several enlarged elastic hard movable lymph nodes were palpable in the region. Salivary flow from his right sub-mandibular gland was poor. Our patient had a history of a recurrent gastric ulcer from 21 years of age. His laboratory data were in the normal range except for lactate dehydrogenase (214 U/L; normal range: 105-210 U/L), γ-glutamyl transpeptidase (88 U/L; normal range: 11-80 U/L), triglyceride (199 mg/dL; normal range: 42-168 mg/dL), leucine aminopeptidase (83 U/L; normal range: 19-69 U/L), and serum immunoglobulin (IgG, 924 mg/dL; normal range: 870-1700 mg/dL). Regarding the measurement of the serum IgG subclass, only his IgG4 level (16.9 mg/dL; normal range: 4.8-105 mg/dL) was evaluated with the consent of our patient. Other serological data regarding the Ig levels and auto-antibodies were not evaluated.

**Figure 1:**
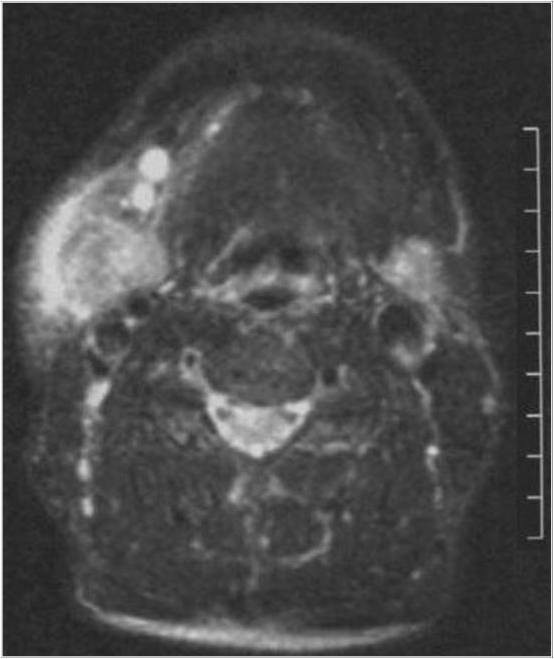
**Fat-saturated T2-weighted weighted MRI**. Fat-saturated T2-weighted weighted MRI showing a heterogeneous hyperintense mass with indistinct margins between the sub-mandibular gland and the lymph nodes. The mass extended invasively to the outer adjacent tissue of the sub-mandibular gland. The sub-mandibular lymph nodes were hyperintense.

Contrast-enhanced computed tomography of the neck revealed a 2.8 × 2.8 cm homogeneously hyper-dense enhanced mass in the right side of his neck which invasively extended to the tissue of his sub-mandibular gland. Several enlarged lymph nodes were enhanced. In the sub-mandibular region, magnetic resonance imaging (MRI) showed a 3.0 × 3.0 × 4.0 cm mass with hypointensity on fat-saturated T1-weighted images, and heterogeneous hyperintensity and mid-hyperintensity on gadolinium-enhanced and fat-saturated T2-weighted images, respectively (Figure 1). Positron emission tomography showed an area of high uptake in the same region. No other uptake lesions were detected. The clinical and radiological appearance suggested a diagnosis of malignant neoplasms of the sub-mandibular gland and metastatic spread from a malignant tumor. Reactive follicular hyperplasia was found on an excisional lymph node biopsy at level II. CD45 gating for routine flow cytometric analysis revealed 40.1% and 27.1% of kappa and lambda light chain expressing cells, respectively.

His right sub-mandibular gland was completely excised along with lymph nodes in the sub-mandibular region. Our patient has been well and there have been no marked changes in his condition at 16 months post-operatively.

**Figure 2:**
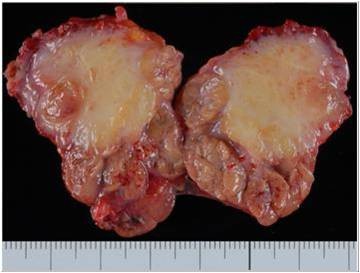
**Photograph of the cut surface of the sub-mandibular gland**. Photograph of the cut surface of the sub-mandibular gland. The sub-mandibular gland space was occupied solid, yellow-wish substances.

**Figure 3:**
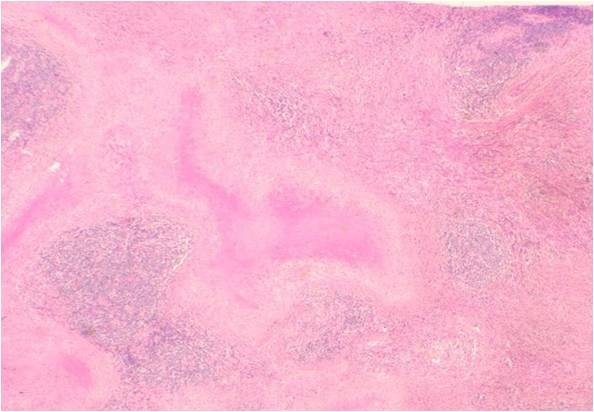
**Photograph of hematoxylin-eosin staining of the excised sub-mandibular gland**. The excised sub-mandibular gland architecture was replaced by prominent fibrocollagenous tissues that exhibited lymphoid follicle-like nodular proliferation (hematoxylin-eosin staining, original magnification ×20).

**Figure 4:**
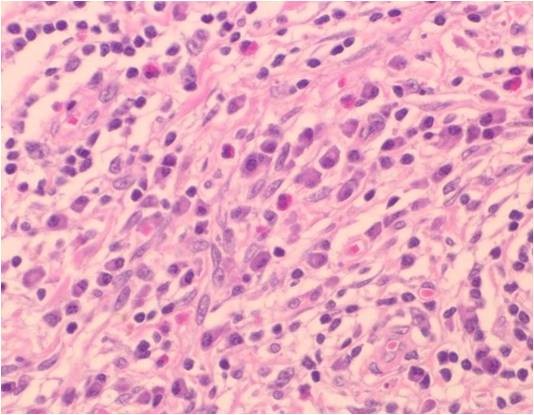
**Photograph of hematoxylin-eosin staining of the excised sub-mandibular gland**. The excised lesion contained spindle cells, scattered lymphocytes, eosinophils and plasma cells (hematoxylin-eosin staining, original magnification ×400).

**Figure 5:**
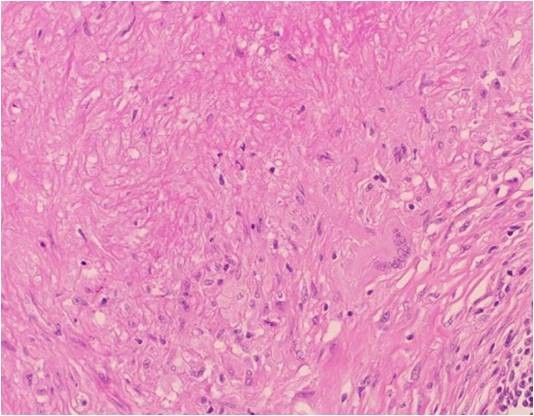
**Photograph of hematoxylin-eosin staining of the excised sub-mandibular gland**. Necrotic tissue with foreign body cells in the excised lesion (hematoxylin-eosin staining, original magnification ×200).

**Figure 6:**
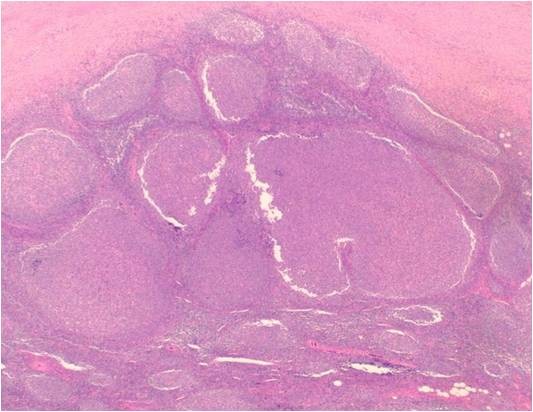
**Photograph of hematoxylin-eosin staining of the excised sub-mandibular gland**. Variously sized and irregularly shaped lymphoid follicle-like nodules proliferated in the excised lesion (hematoxylin-eosin staining, original magnification ×40).

**Figure 7:**
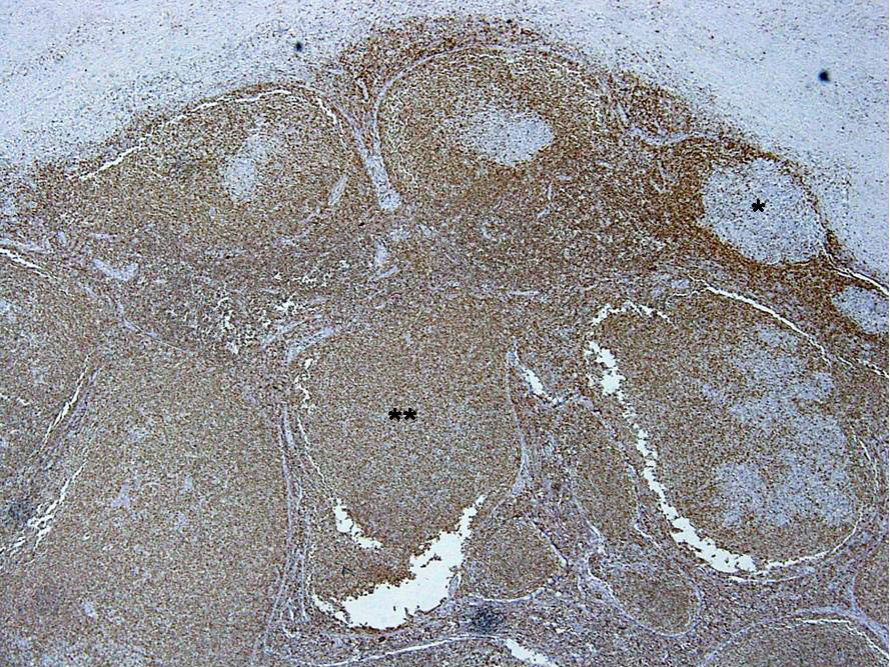
**Photograph of bcl-2 staining of the excised sub-mandibular gland**. Photograph of Bcl-2 staining of the excised lymph node, with the same field of view as in Figure 6. High-power magnification showing reactive germinal centers of the lymphoid follicles negative for Bcl-2 (*), but nodular lesions lacking definitive germinal centers and mantle zones positive for Bcl-2 (**) (original magnification ×40).

**Figure 8:**
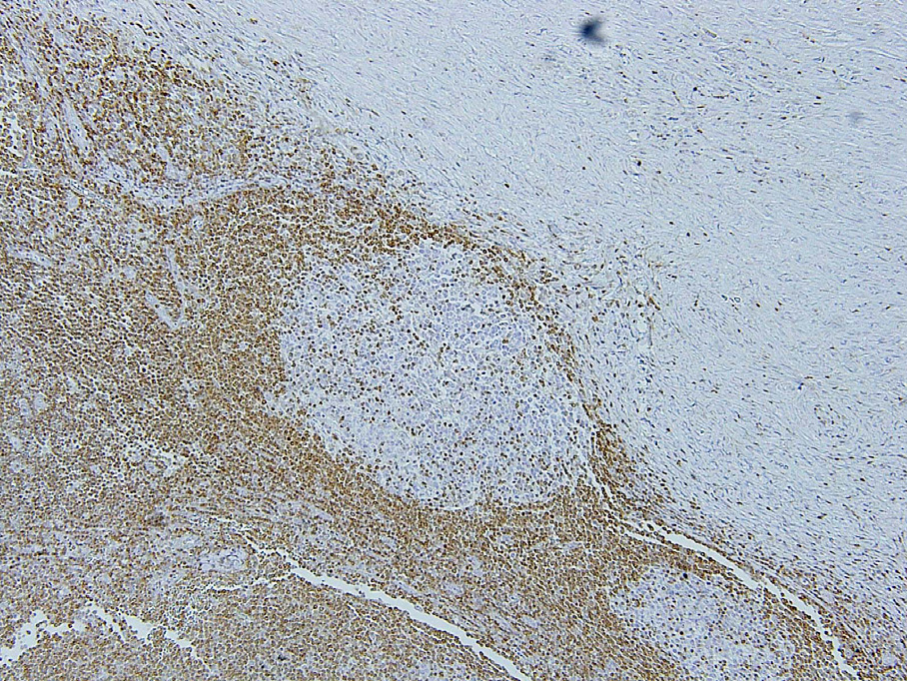
**Photograph of bcl-2 staining of the excised sub-mandibular gland**. High-power magnification of the lesion marked by the asterisk in Figure 7 (original magnification ×100).

**Figure 9:**
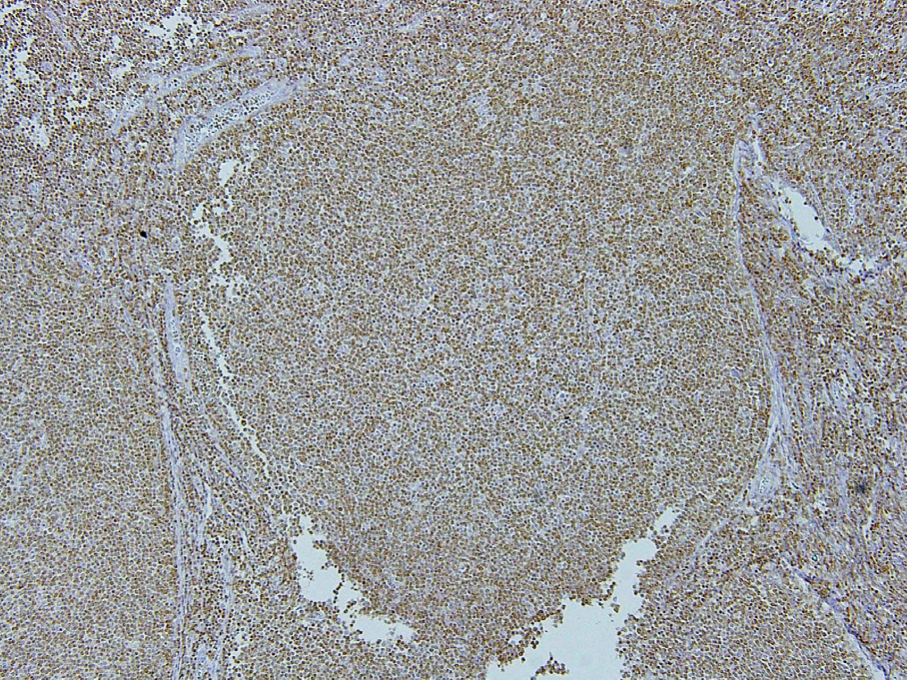
**Photograph of bcl-2 staining of the excised sub-mandibular gland**. High-power magnification of the lesion marked by the double asterisks in Figure 7 (original magnification ×100).

**Figures 10:**
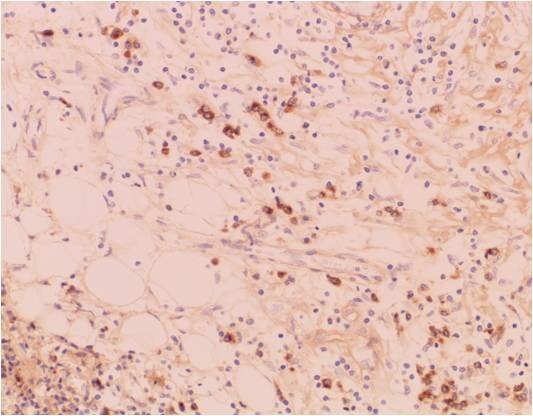
**Photographs of IgG staining of the excised sub-mandibular gland**. Photograph of IgG staining of the excised sub-mandibular gland (original magnification ×200).

**Figures 11:**
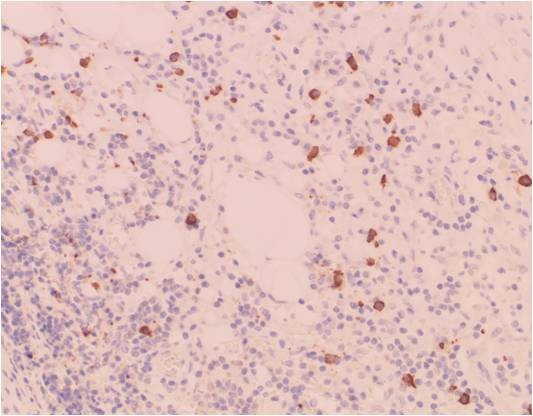
**Photographs of IgG4 staining of the excised sub-mandibular gland**. Photograph of IgG4 staining of the excised sub-mandibular gland (original magnification ×200).

*Pathological and immunohistological findings *The sub-mandibular gland: The cut surface of the surgical specimen showed a solid, yellowish tumor occupying the sub-mandibular gland space (Figure 2). At low magnification, marked parenchymal loss with severe fibrocollagenous changes and numerous inflammatory cells were noted (Figure 3). At higher magnification, scattered lymphocytes, eosinophils and plasma cells were observed in the peri-ductal fibrosis area (Figure 4). A few foreign body cells were noted in necrotic tissue (Figure 5). No EBER-positive cells were seen, and no acid-fast bacilli were detected by Ziehl-Neelsen staining. No lympho-epithelial lesions were detected. In the fibrosis area, small and large-sized lymphoid follicle-like nodular lymphocytic proliferations were observed (Figure 6). Paraffin-embedded tissue sections, fixed in formalin, were stained with the antibodies listed in Table 1. An lmmunohistochemical study revealed that the lymphoid follicle-like nodular lesions were occupied by small round to oval shaped lymphocytes. These cells tested positive for CD20 and Bcl-2 (Figure 7, Figure 8, Figure 9), and negative for CD3, CD5, CD10 and cyclinD1. IgG4-positive plasma cells infiltration was observed. On a highly magnified slide checked at five points, 30% of IgG positive plasma cells expressed IgG4 (Figure 10, Figure 11).

**Table 1:** Primary antibodies used in this study

Antigen	Supplier	Clone name	Dilution
CD3	Novocastra	NCL-CD3-PS1	1: 200
CD5	MBL	4C7	1: 200
CD10	Novocastra	NCL-CD10-270	1: 100
CD20	Dako	L26	1: 500
Bcl-2	Dako	124	1: 50
CyclinD1	Nichirei	Sp4	1: 25
IgG	Dako	N1508	1: 8
IgG4	Binding Site Ltd.	PC009	1: 5000

DakoCytomation, Glostrup, Denmark; MBL, Nagoya, Japan; Nichirei, Tokyo, Japan; Binding Site Ltd., Birmingham, UK

**Figures 12:**
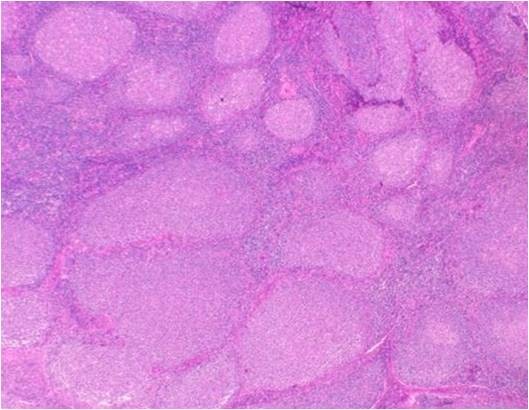
**Photograph of hematoxylin-eosin staining of the excised lymph node**. The lymph node architecture was replaced by prominent fibrocollagenous tissues and irregularly shaped lymphoid follicle-like nodules proliferated (original magnification ×20).

**Figure 13:**
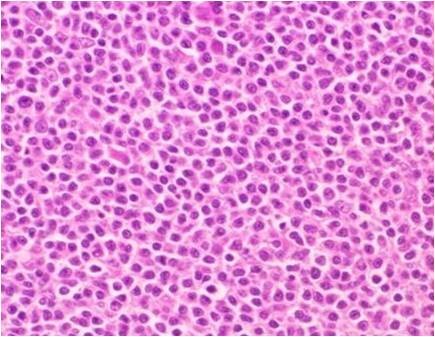
**Photograph of hematoxylin-eosin staining of the excised lymph node**. Small round to oval cells proliferated in the irregularly shaped lymphoid follicle-like node (original magnification ×400).

The lymph nodes: The lymph node architecture was replaced by prominent fibrocollagenous tissue with scattered lymphocytes and plasma cells. Except for lymphoid follicles with a reactive germinal center, small and large-sized lymphoid follicle-like lymphocytic proliferations were observed. These nodular lesions lacked a definitive germinal center and mantle zone (Figure 12), and were occupied by small round to oval cell lymphocytes (Figure 13). These cells were positive for CD20 and Bcl-2, and negative for CD3, CD5, CD10 and cyclinD1.

## Discussion

The histological and cytological features of Küttner's tumor show various characteristics, according to stage in the progressive process and severity of inflammation [1]. According to Seifert [1], Küttner's tumor may evolve through four different histological stages as follows:

*Stage 1*: Focal chronic inflammation with nests of lymphocytes around salivary ducts, which are moderately dilated and contain inspissated secretion.

*Stage 2*: More marked diffuse lymphocytic infiltration, and more severe peri-ductal fibrosis. The ductal system shows inspissated secretion and focal metaplasia with proliferation of ductal epithelium. Peri-ductal lymphoid follicles are well developed. There is fibrosis in the centers of the lobules, accompanied by atrophy of acini.

*Stage 3*: Even more prominent lymphocytic infiltration, with lymphoid follicle formation, parenchymal atrophy, peri-ductal hyalinization, and sclerosis. Squamous and goblet cell metaplasia in the ductal system.

*Stage 4 (end-stage)*: Cirrhosis-like, with marked parenchymal loss and sclerosis (the "burnt out" phase).

Our case corresponded to stage 4. The morphological and histological findings presented important differential diagnostic problems between the sclerosing variant of follicular lymphoma involving the sub-mandibular glands, and Küttner's tumor associated with lymphoid hyperplasia of the regional lymph nodes. In the former, lymphoid follicles are separated by bands of collagenized stroma and are composed of relatively poorly defined germinal centers, with a narrow mantle zone. Immunohistochemical study reveals the presence of monoclonal B cells, CD10 and Bcl-2 positivity [4]. Non-neoplastic reactive germinal centers contain variously sized cells such as centrocyte-like and centroblast-like, medium-sized lymphocytes, and Bcl-2 is expressed in the mantle cells [5]. In non-neoplastic germinal center cells, Bcl-2 is absent [6]. In our case, however, B-cells of similar size and shape occupied the lymphoid follicle-like nodules and stained positive for Bcl-2. These findings therefore suggested unusual B-cell proliferation. Indeed, unusual B-cells were detected as polyclonal B-cells by flow cytometric analysis in our case, and were negative for CD10.

We interpret the morphological and immunophenotypic features of lymphoid follicle-like nodules of our case to be similar to follicular hyperplasia, which is characterized by the presence of progressive transformation of the germinal centers. In such progressive transformation, it is thought that small mantle B-lymphocytes invade and sequentially replace the reactive germinal centers and, as a result, the follicle has no evident mantle zone [5] and the follicular center cells express Bcl-2 [7].

The typical findings of extra-nodal marginal zone B-cell lymphoma of mucosa-associated lymphoid tissue (MALT lymphoma) were not detected in our case. Some cases of Küttner's tumor need to be distinguished from MALT lymphoma, where the neoplastic cells are post-germinal center B-cell lymphocytes, which are slightly larger than normal small lymphocytes and have a centrocyte-like or monocytoid appearance. Some hyperplastic ductal epithelium persists and is permeated by neoplastic lymphocytes, so-called lympho-epithelial lesions [8].

Although unusual B-cell proliferation was seen in our case, no definite evidence of B-cell lymphoma was found and so the lesion was diagnosed as Küttner's tumor associated with fibrosclerosis and follicular hyperplasia of the regional lymph nodes. Some case reports of lymphomas with a background of autoimmune disease or chronic inflammation appear in the literature [9-11]. The consent of our patient was not received for additional testing, such as a southern blot, which made it difficult to investigate all the diagnostic problems in detail. However, we have strictly structured follow-up in order to monitor whether our patient shows neoplastic transformation; he has been well to date with no recurrence.

Our case showed cervical lymphadenopathy, therefore sarcoidosis [12], tuberculosis [13,14], Kimura's disease and plasma cell type of Castleman's disease [3] needed to be excluded in differential diagnosis. We did not detect multiple intra-glandular non-caseating granuloma as appears in sarcoidosis and tuberculosis. There were also no findings indicative of Kimura's disease, namely, eosinophilic lymphofolliculoid granuloma with lymphoid hyperplasia, remarkable infiltration of eosinophils and proliferation of capillaries [15]. The clinical and pathological findings also enabled us to exclude plasma cell type of Castleman's disease. Bowne *et al*. note that the diagnosis must be considered in the appropriate clinical setting only after the more common causes of lymphoid adenopathy have been investigated and excluded, because patients with Castleman's disease frequently show systemic symptoms and abnormal laboratory findings such as anemia, fever, fatigue, hypoalbuminemia and hyperglobulinemia [16].

Kitagawa *et al*. suggested that dense infiltration of IgG4-positive plasma cells detected in the salivary glands with sclerosing sialadenitis is indicative of Küttner's tumor [2]. Patients with an IgG4-related idiopathic sclerosing lesion frequently have regional lymph adenopathy [1-3]. In Japan, diagnosis of IgG4-related disease is defined by both elevated serum IgG4 (>135 mg/dl) and histopathological features including lymphocyte and IgG4(+) plasma cell infiltration (IgG4(+) plasma cells/IgG(+) plasma cells >50% on a highly-magnified slide checked at five points). Our case did not meet these criteria. Kojima *et al*. suggested that a possible relationship between IgG4-related idiopathic sclerosing lesion and lymph nodal adenopathy has been rarely discussed because little is known about the histopathological and immunohistochemical findings [3].

In our case, marked fibrosclerosis was observed in both the sub-mandibular gland and regional lymph nodes. We could not detect a definitive diagnostic factor. Further studies are needed to clarify the etiology of idiopathic sclerosing lesion associated with a lymphoid lesion arising in both the sub-mandibular gland and regional lymph nodes.

## Conclusion

We have reported a rare case of Küttner's tumor associated with fibrosclerosis and atypical lymphoid hyperplasia in both the sub-mandibular gland and regional lymph nodes. To clarify the pathogenic mechanism of such idiopathic sclerosing lesions, more cases need to be investigated. However, our information might be helpful to further studies seeking to clarify the etiology of idiopathic sclerosing lesions arising in the organs and regional lymph nodes.

## Consent

Written informed consent was obtained from the patient for publication of this case report and any accompanying images. A copy of the written consent is available for review by the Editor-in-Chief of this journal.

## Competing interests

The authors declare that they have no competing interests.

## Authors' contributions

YM drafted the manuscript and described the pathology component. KO edited the clinical part of the manuscript. KK, KS and YA made the final histopathological diagnosis and revised the manuscript for important intellectual content. HS provided the clinical data. All authors have read and approved the final manuscript.
